# TDP-43 seeding activity in the olfactory mucosa of patients with amyotrophic lateral sclerosis

**DOI:** 10.1186/s13024-025-00833-0

**Published:** 2025-04-26

**Authors:** Maria Vizziello, Ilaria Linda Dellarole, Arianna Ciullini, Riccardo Pascuzzo, Annalisa Lombardo, Floriana Bellandi, Luigi Celauro, Claudia Battipaglia, Emilio Ciusani, Ambra Rizzo, Marcella Catania, Grazia Devigili, Sara Adriana Della Seta, Valentina Margiotta, Monica Consonni, Veronica Faltracco, Pietro Tiraboschi, Nilo Riva, Sara Maria Silvia Portaleone, Gianluigi Zanusso, Giuseppe Legname, Giuseppe Lauria, Eleonora Dalla Bella, Fabio Moda

**Affiliations:** 1 Department of Pharmacological and Biomolecular Sciences, University of Milan, Milan, Italy; 2 Unit of Neurology 3 – Neuroalgology and Motor Neuron Disease Centre, Fondazione IRCCS Istituto Neurologico Carlo Besta, Milan, Italy; 3 Unit of Neurology 5 – Neuropathology, Fondazione IRCCS Istituto Neurologico Carlo Besta, Milan, Italy; 4 Neuroradiology Unit, Fondazione IRCCS Istituto Neurologico Carlo Besta, Milan, Italy; 5 Laboratory of Prion Biology, Department of Neuroscience, Scuola Internazionale Superiore Di Studi Avanzati (SISSA), Trieste, Italy; 6 Unit of Laboratory Medicine, Fondazione IRCCS Istituto Neurologico Carlo Besta, Milan, Italy; 7 Unit of Neurology 1 – Parkinson and Movement Disorders, Fondazione IRCCS Istituto Neurologico Carlo Besta, Milan, Italy; 8 Department of Health Sciences, Otolaryngology Unit, ASST Santi Paolo E Carlo Hospital, Università Degli Studi Di Milano, Milan, Italy; 9 Department of Neurosciences, Biomedicine and Movement Sciences, University of Verona, Verona, Italy; 10 Scientific Directorate, Fondazione IRCCS Istituto Neurologico Carlo Besta, Milan, Italy; 11 Department of Medical Biotechnology and Translational Medicine, University of Milan, Milan, Italy

**Keywords:** TDP-43, Olfactory mucosa, Seed amplification assay, Amyotrophic lateral sclerosis, Peripheral biomarkers, Neurodegeneration

## Abstract

**Background:**

In recent years, the seed amplification assay (SAA) has enabled the identification of pathological TDP-43 in the cerebrospinal fluid (CSF) and olfactory mucosa (OM) of patients with genetic forms of frontotemporal dementia (FTD) and amyotrophic lateral sclerosis (ALS). Here, we investigated the seeding activity of TDP-43 in OM samples collected from patients with sporadic ALS.

**Methods:**

OM samples were collected from patients with (a) sporadic motor neuron diseases (MND), including spinal ALS (*n* = 35), bulbar ALS (*n* = 18), primary lateral sclerosis (*n* = 10), and facial onset sensory and motor neuronopathy (*n* = 2); (b) genetic MND, including carriers of *C9orf72*^*exp*^ (*n* = 6), *TARDBP* (*n* = 4), *SQSTM1* (*n* = 3), *C9orf72*^*exp*^ + *SQSTM1* (*n* = 1), *OPTN* (*n* = 1), *GLE1* (*n* = 1), *FUS* (*n* = 1) and *SOD1* (*n* = 4) mutations; (c) other neurodegenerative disorders (OND), including Alzheimer’s disease (*n* = 3), dementia with Lewy bodies (*n* = 8) and multiple system atrophy (*n* = 6); and (d) control subjects (*n* = 22). All samples were subjected to SAA analysis for TDP-43 (TDP-43_SAA). Plasmatic levels of TDP-43 and neurofilament-light chain (NfL) were also assessed in a selected number of patients.

**Results:**

TDP-43_SAA was positive in 29/65 patients with sporadic MND, 9/21 patients with genetic MND, 6/17 OND patients and 3/22 controls. Surprisingly, one presymptomatic individual also tested positive. As expected, OM of genetic non-TDP-43-related MND tested negative. Interestingly, fluorescence values from non-MND samples that tested positive were consistently and significantly lower than those obtained with sporadic and genetic MND. Furthermore, among TDP-43-positive samples, the lag phase observed in MND patients was significantly longer than that in non-MND patients. Plasma TDP-43 levels were significantly higher in sporadic MND patients compared to controls and decreased as the disease progressed. Similarly, plasma NfL levels were higher in both sporadic and genetic MND patients and positively correlated with disease progression rate (ΔFS). No significant correlations were detected between TDP-43_SAA findings and the biological, clinical, or neuropsychological parameters considered.

**Conclusions:**

The OM of a subset of patients with sporadic MND can trigger seeding activity for TDP-43, as previously observed in genetic MND. Thus, TDP-43_SAA analysis of OM can improve the clinical characterization of ALS across different phenotypes and enhance our understanding of these diseases. Finally, plasma TDP-43 could serve as a potential biomarker for monitoring disease progression. However, further research is needed to confirm and expand these findings.

**Supplementary Information:**

The online version contains supplementary material available at 10.1186/s13024-025-00833-0.

## Background

Amyotrophic lateral sclerosis (ALS) is a fatal neurodegenerative disorder characterized by progressive motor decline resulting from the loss of upper and lower motor neurons (UMNs and LMNs, respectively). The median survival for classic ALS is 2–3 years from symptom onset, although survival varies widely across different disease forms [1, 2]. ALS presents with distinct clinical subtypes: (1) spinal-onset ALS (sALS), affecting the limbs with both UMN and LMN involvement (~ 70–75% of cases); (2) bulbar-onset ALS (bALS), leading to speech and swallowing difficulties (~ 20–25% of cases); (3) primary lateral sclerosis (PLS), presenting with pure UMN involvement; (4) progressive muscular atrophy (PMA), with pure LMN involvement [3, 4]; and (5) respiratory-onset ALS (rALS, 3–5% of cases) [5]. Facial onset sensory and motor neuronopathy (FOSMN) is a rare disorder considered part of the ALS spectrum [6]. Cognitive and behavioral symptoms, ranging from mild impairment to full-blown dementia syndrome fulfilling the criteria for frontotemporal dementia (FTD) [7], further contribute to ALS heterogeneity. Survival greatly varies by phenotype, with PLS and PMA typically allowing for longer survival [8]*,* while bALS and rALS are associated with poorer prognosis [4, 9]. Despite clinical heterogeneity, nearly all ALS cases share a key pathological signature: cytoplasmic inclusions of misfolded transactive response DNA-binding protein 43 kDa (TDP-43) in degenerating neurons [10, 11]. Therefore, TDP-43 aggregates are a hallmark of ALS, and their detection in postmortem brain and spinal cord tissue enables a definitive diagnosis [10, 12]*.* However, TDP-43 pathology is also observed in other neurodegenerative diseases, including frontotemporal lobar degeneration with TDP-43-immunoreactive pathology (FTLD-TDP) [13], Perry disease [14], limbic-predominant age-related TDP-43 encephalopathy (LATE) [15], sporadic inclusion body myositis (sIBM) [16], Alzheimer’s disease (AD) and hippocampal sclerosis [17], Parkinson’s disease (PD) [18], dementia with Lewy bodies (DLB) [19], multiple system atrophy (MSA) [20], corticobasal degeneration (CBD) [21], progressive supranuclear palsy (PSP) [22], and Huntington’s disease (HD) [23]. Additionally, TDP-43 deposition has been reported in the brains of healthy elderly individuals over 90 years of age [24]. TDP-43 is a ubiquitously expressed DNA/RNA-binding protein encoded by the *TARDBP* gene (chromosome 1p36.22) [25]*.* The protein is normally localized in the nucleus of neuronal and glial cells, and it continuously shuttles into the cytoplasm in a transcription-dependent manner. The tight self-regulation of its steady-state levels is crucial for the maintenance of cellular homeostasis [26, 27]. Under pathological conditions, TDP-43 misfolds and aggregates in the cytosol of both neurons and glial cells, becoming aberrantly phosphorylated, ubiquitinated, and cleaved to generate truncated C-terminal fragments (CTFs) [10, 28, 29]. Interestingly, the composition of neuronal cytoplasmic inclusions (NCIs) shows regional differences, with cortical and hippocampal NCIs being enriched in CTFs, whereas NCIs in the spinal cord display a greater proportion of full-length TDP-43, suggesting that regionally distinct pathogenic processes may underlie inclusion body formation in TDP-43 pathology [30, 31]*.* TDP-43 can undergo different types of misfolding, acquiring various aberrant conformations that, like prions and other prion-like proteins, are called strains. These strains can lead to different TDP-43 proteinopathies (e.g. FTD vs. ALS) or be responsible for different phenotypes of the same disease (e.g. bulbar vs. spinal ALS), thereby contributing to the phenotypic heterogeneity of these conditions [32–41]. Approximately 90% of ALS cases are sporadic, while 10% have a family history, mostly with autosomal dominant inheritance with variable penetrance [42]. TDP-43 pathology is present in most sporadic cases and in genetic ALS associated with mutations in chromosome 9 open reading frame 72* (C9orf72), TARDBP*, optineurin (*OPTN), *sequestosome 1* (SQSTM1), *ubiquilin- 2* (UBQLN2), *and TANK binding kinase 1* (TBK1)* [43]. Another rare mutation in Nucleoporin (*GLE1* RNA export mediator) appears to be associated with ALS, and several studies are currently underway to confirm this link [44]. However, familial ALS caused by superoxide dismutase type 1 (*SOD1*) and fused in sarcoma (*FUS*) mutations typically lack TDP-43 pathology [45–47]*.* ALS diagnosis currently relies on clinical and neurophysiological findings, but disease-specific biomarkers are urgently needed for early diagnosis, patient stratification, prognosis and therapeutic development. TDP-43 protein is a promising candidate due to its presence in peripheral biofluids, cells and tissues beyond the central nervous system (CNS). A very recent study revealed that plasma levels of TDP-43 are significantly higher in ALS patients than in controls, supporting its potential as a blood-based biomarker for the diagnosis of TDP-43 proteinopathies [48]. Reduced levels of TDP-43 were found in the serum of genetic FTD patients with *C9orf72* expansion or FTD with motor neuron disease (FTD-MND) [49]. Another recent publication showed that plasma extracellular vesicles (EVs) contain measurable levels of TDP-43 and tau isoforms, which can effectively differentiate between TDP-43 and tau pathologies in FTD and ALS patients, with high diagnostic accuracy [50]. Remarkably, several autopsy studies have shown the presence of TDP-43 aggregates in the olfactory bulb of patients with ALS [51]. In recent years, seed amplification assays (SAAs) have been developed to detect tiny amounts of pathological proteins in peripheral tissues of patients with various neurodegenerative diseases, including prion diseases, α-synucleinopathies, tau and TDP-43 proteinopathies [52–55]. Interestingly, SAAs have successfully detected pathological TDP-43 in the cerebrospinal fluid (CSF) and olfactory mucosa (OM) of patients with genetic forms of ALS and FTD associated with TDP-43 pathology [54, 55]. In this study, we applied SAA to OM samples from patients with sporadic forms of motor neuron disease (MND) within the ALS spectrum to assess their ability to seed TDP-43 aggregation. We also evaluated their α-synuclein seeding activity via α-synuclein SAA (αSyn_SAA) and measured plasma levels of TDP-43 and neurofilament-light chain (NfL), aiming to explore their potential as biomarkers for ALS.

## Methods

### Study participants

A consecutive cohort of 86 patients diagnosed with sporadic and genetic ALS according to the revised El Escorial criteria [56] was recruited from outpatients and inpatients referred to the Motor Neuron Diseases Centre of the Fondazione IRCCS Istituto Neurologico Carlo Besta (Milan, Italy) between December 2022 and January 2025. The enrolled patients were affected by different disease variants (Table 1). Seventeen patients with other neurodegenerative diseases (ONDs) and 22 healthy controls (CTRLs) were also included. A detailed list of the enrolled subjects is reported in Table 1. We would like to clarify that in this manuscript, the acronym sALS refers to spinal-onset ALS rather than sporadic forms. Written informed consent for the use of anonymized clinical data for research purposes was obtained at the time of evaluation from all the subjects included in the analysis.

**Table 1:** List of subjects enrolled in the study

**Clinical Diagnosis**	**OM samples (number)**	**Age, mean (SD) (y)**	**Sex (M/F)**	**Age at onset, mean (SD) (y)**	**Disease duration, mean (SD) (m)**	**ALSFRS-R, mean (SD; range)**	**Progression rate, mean (SD)**	**Cognitive phenotype** **(ALScn/ALSimp/FTD)**	**Onset region (number; spinal/bulbar)**
**Sporadic MND**	**65**	62.9 (10.1)	48/17	61 (10.3)	28 (27.4)	39.9 (5.5;19–47)	1.0 (0.6)	34/21/10	44/21
sALS	35	62.1 (9.9)	27/8	61 (10.3)	20 (24.7)	39.6 (6.3;19–47)	0.8 (0.8)	22/9/4	35/0
bALS	18	63.8 (10.0)	11/7	61 (9.8)	28 (22.1)	40.6 (4.0;32–46)	0.36 (0.3)	9/6/3	0/18
PLS	10	62.9 (10.1)	9/1	59 (10.4)	47 (27.4)	NA	NA	3/5/2	9/1
FOSMN	2	70.0 (0)	1/1	64 (6.4)	78 (78.5)	NA	NA	0/1/1	0/2
**Genetic MND**	**21**	58.9 (10.1)	10/11	55 (10.7)	46 (34.1)	38.6 (5.5;18–46)	0.38 (0.4)	15/1/2 §	14/6
*C9orf72*^*exp*^	6 (1 presymptomatic)	60.0 (9.8)	4/2	59 (10.2)	30 (26.4)	40.2 (5.9;37–44)	0.4 (0.7)	4/1/1	3/2
*TARDBP*	4	53.8 (10.2)	1/3	50 (10.4)	38 (18.8)	40.5 (4.0;34–46)	0.25 (0.7)	4/0/0	3/1
*SQSTM1*	3	70.0 (10.5)	1/2	66 (10.7)	51 (20.0)	39.3 (5.4;35–43)	0.29 (0.8)	0/0/1 §	2/1
*C9orf72*^*exp*^ + *SQSTM1*	1	57	1/0	57	22	18	1.36	1/0/0	1/0
*OPTN*	1	58	1/0	53	59	NA	NA	1/0/0	1/0
*GLE1*	1	65	0/1	64	7	39	1.29	1/0/0	0/1
*FUS*	1	49	1/0	47	17	44	0.24	1/0/0	1/0
*SOD1*	4	55 (5.1)	1/3	48 (4.8)	92 (28.3)	38.0 (4.0; 36–44)	0.11 (0.05)	3/0/0 §	4/0
**Other neurodegenerative diseases**	**17**	67.4 (9.0)	14/3	NA	NA	NA	NA	NA	NA
MSA	6	63.3 (8.1)	4/2	NA	NA	NA	NA	NA	NA
DLB	8	69.0 (8.7)	8/0	NA	NA	NA	NA	NA	NA
AD	3	71.0 (11.8)	2/1	NA	NA	NA	NA	NA	NA
**CTRLs**	**22**	51.9 (16.8)	11/11	NA	NA	NA	NA	NA	NA
**TOTAL**	**121**	61.1 (12.3)	82/39	NA	NA	NA	NA	NA	NA

Abbreviations: OM = olfactory mucosa; SD = standard deviation; y = years; M = male; F = female; m = months; ALS-FRS-R = ALS Functional Rating Scale Revised; ALScn: cognitively normal ALS; ALSimp = ALS with cognitive impairment (including ALSci [ALS with cognitive impairment], ALSbi [ALS with behavioral impairment], ALScbi [ALS with combined cognitive and behavioral impairment], ALSnex [ALS with nonexecutive dysfunction]); ALS-FTD: ALS with frontotemporal dementia; MND = motor neuron disease; sALS = spinal-onset amyotrophic lateral sclerosis; bALS = bulbar-onset amyotrophic lateral sclerosis; PLS = primary lateral sclerosis; FOSMN = facial onset sensory and motor neuronopathy; *C9orf72exp* = chromosome 9 open reading frame 72 expansion; *TARDBP* = TAR DNA-binding protein; *SQSTM1* = sequestosome 1; *OPTN* = optineurin; *GLE1* = GLE1 RNA export mediator; *FUS* = fused in sarcoma; *SOD1* = superoxide dismutase type 1; MSA = multiple system atrophy; DLB = dementia with Lewy body; AD = Alzheimer's disease; CTRLs = controls; NA = not assessed

^§^ Neuropsychological data are not available for three patients (2 *SQSTM1*, and 1 *SOD1*)

### Clinical, neuropsychological and genetic assessment

Neurologists with expertise in motor neuron diseases collected familial and clinical history and performed neurological examinations of all patients who underwent OM sample collection. The following basic epidemiological and clinical data were collected: sex, age at onset, disease duration at evaluation, age at evaluation, involved body region(s) at onset, and family history of ALS/FTD or other neurodegenerative and neuropsychiatric disorders. Patients diagnosed with sporadic MND (*n* = 65) were distinguished according to their motor phenotype (sALS, 35; bALS, 18; PLS, 10; and FOSMN, 2). Patients diagnosed with genetic MND (*n* = 21) were distinguished according to their motor phenotype (sALS, 13; bALS, 6; PLS, 1; presymptomatic carrier, 1) and genotype (*C9orf72*^*exp*^, 6; *TARDBP*, 4; *SQSTM1*, 3; *C9orf72*^*exp*^ + *SQSTM1*, 1; *OPTN*, 1; *GLE1*, 1; *FUS*, 1; *SOD1*, 4). For patients with sALS or bALS, motor functional disabilities were measured via the revised version of the ALS Functional Rating Scale (ALSFRS-R) [57], and the disease progression rate (ΔFS) was calculated according to the following formula: (48 – ALSFRS-R score)/disease duration at evaluation (months) [58]. Clinical staging was estimated according to King’s Clinical Staging System [59]*.* All patients underwent extensive neurophysiological evaluation by qualified physicians according to established standards and a comprehensive and standardized neuropsychological test battery. The Italian version of the Edinburgh Cognitive and Behavioral ALS Screen (ECAS) has been used as a multidomain neuropsychological screening tool specifically designed to account for difficulties with speech, writing, and drawing [60]. The ECAS assesses the following cognitive domains: executive functions, social cognition, verbal fluency, and language (ALS-specific), as well as memory and visuospatial abilities (non-ALS-specific). For statistical purposes, we used ECAS scores corrected for age and education on the basis of Italian regression-based norms [61]. Patients with cognitive (ALSci), behavioral (ALSbi), combined cognitive and behavioral (ALScbi) and/or frontotemporal dementia-like (ALS-FTD) impairments were defined according to recent guidelines [62]. We designated patients with isolated nonexecutive cognitive dysfunction in the domains of memory and visuospatial function as ALSnex patients. In addition, all patients also underwent blood sampling for routine blood tests and genetic screening [63]. Targeted next-generation sequencing (NGS) analysis was performed in all patients via amplicon deep sequencing via the Sure Select QXT Kit (Agilent) for ALS-causative genes (*SOD1*, *FUS*, *TARDBP*, *VCP*, *PFN1*, *TUBA4 A*, *OPTN*, *SQSTM1*, *UBQLN2*), and a repeat primed PCR AmplideX PCR/CE C9ORF72 Kit (Asuragen Inc., Austin TX) for the detection of *C9orf72* expansion was used in all patients. Selected patients underwent more extensive genetic analysis.

### OM samples collection and preparation for SAA analyses

OM samples were obtained by a specialized otolaryngologist using a fiberoptic rhinoscope to locate the olfactory mucosa. The medial septal wall, just above the middle turbinate of both nostrils, was gently brushed with a special flocked swab (FLQBrushTM Copan Italia, Brescia, Italy), as previously described [64, 65]. After collection, the swabs were immersed in 9 mL of saline solution, vortexed and then centrifuged at 800 × g for 20 min at 4 °C. For TDP-43_SAA, 2 μg of the pellet was collected using an inoculating loop and transferred into a tube with 25 μL of PBS. This sample was then further diluted 1:10 in PBS before TDP-43_SAA analysis. For αSyn_SAA, 2 μg of the pellet was resuspended in 17 μL of PBS, and 2 μL of this mixture was further diluted in 38 μL of PBS with 1% Triton X-100 and used for SAA analysis.

### Recombinant TDP-43 production

The truncated construct (HuTDP-43(263–414)) was obtained using mutagenesis by deleting the DNA region encoding the HuTDP-43 fragment 1–262 from the pET- 11a plasmid containing human TDP-43 (HuTDP-43(1–414)) (GenScript). The primers used in the polymerase chain reaction (site-directed mutagenesis kit (Agilent)) were as follows: 5′-ttaagaaggagatatacatatgaaacacaacagcaaccgtcagc- 3′ and 5′-gctgacggttgctgttgtgtttcatatgtatatctccttcttaa- 3′. *Escherichia coli* BL21 (DE3) cells (Stratagene) were used to express the truncated construct, and the transformed culture was conditioned with fresh medium (Luria Bertani) supplemented with 100 μg/mL ampicillin. When the culture reached an OD600 of 0.8, protein expression was triggered with 1 mM isopropyl β-d-1-thiogalactopyranoside. After incubation overnight at 37 °C, the cells were lysed via a homogenizer (PandaPLUS 2000) in buffer containing 25 mM Tris–HCl, 5 mM ethylenediaminetetraacetic acid (EDTA), and 0.8% Triton X-100 (pH 8), and the inclusion bodies were washed multiple times in bidistilled water (ddH2O). The inclusion bodies containing HuTDP-43(263–414) were dissolved in five volumes of 8 M guanidine hydrochloride (GdnHCl), loaded onto a preequilibrated HiLoad 26/60 Superdex 200-pg column, and eluted in 25 mM Tris–HCl (pH 8), 5 mM EDTA and 5 M GdnHCl at a flow/rate of 1.5 mL/min. Protein refolding was performed by dialysis using 25 mM Tris–HCl (pH = 8) with a spectrophotometer.

### TDP-43_SAA of the OM samples

TDP-43_SAA analyses were performed following the protocol already described [55], with some modifications. Briefly, 96-well optical flat bottom plates (Thermo Scientific) were used, with each sample tested in triplicate. Each well was filled with 96 μL of TDP-43_SAA reaction mixture composed of HuTDP-43(263–414) [0.05 mg/mL], Tris–HCl (pH 8.0) [40 mM], guanidine hydrochloride (GdnHCl) [500 mM], 0.002% sodium dodecyl sulfate (SDS), and thioflavin-T (ThT) [10 μM]. A 3-mm glass bead (Sigma) and 4 μL of OM sample were added to each well. The plate was then sealed with sealing film (Thermo Scientific), placed into a FLUOstar CLARIOSTAR microplate reader (BMG Labtech) and subjected to alternating cycles of shaking (15 s, 100 rpm, double orbital) and incubation (30 min, 42 °C). ThT fluorescence intensities, expressed as arbitrary units (AUs), were recorded every 30 min at wavelengths of 450 ± 10 nm (excitation) and 480 ± 10 nm (emission), with a bottom read. To ensure assay reliability, each TDP-43_SAA analysis included in-house reaction controls: OM samples from a control subject spiked with in vitro generated aggregates of recombinant HuTDP-43(263–414) as the positive control, and OM spiked with buffer alone as the negative control. The test was considered valid only if these controls performed as expected. To maintain clarity in data presentation, we did not include these control results in the manuscript graphs.

### αSyn_SAA of the OM samples

αSyn_SAA analyses were performed using 384-well optical flat-bottom plates (Thermo Scientific), with each sample tested in triplicate. Each well was filled with 49 μL of αSyn_SAA reaction mixture containing rec-αSyn (purchased from rPeptide, S-1001–2) [0.1 mg/mL], HEPES (pH 8.0) [40 mM], sodium citrate [170 mM], and thioflavin-T (ThT) [20 μM]. Two low-binding silica beads (0.8 mm, OPS Diagnostics) and 1 μL of OM samples were added to each well. The plate was then sealed with sealing film (Thermo Scientific), placed into a FLUOstar OMEGA microplate reader (BMG Labtech) and subjected to alternating cycles of shaking (1 min, 600 rpm, single orbital) and incubation (1 min, 42 °C). ThT fluorescence intensities, expressed as AUs, were recorded every 30 min at wavelengths of 450 ± 10 nm (excitation) and 480 ± 10 nm (emission), with a bottom read.

### Kinetic curve analysis

A two-step preprocessing analysis of the kinetic curves was performed prior to the extraction of quantitative parameters related to the seeding activity of the samples. First, the Hampel filter, a robust method for detecting and handling outliers, was applied to the raw data points for each replicate [66]. This filter consists of a sliding window within which the central point is considered an outlier if its value is greater than 3 standard deviations (estimated through the median absolute deviation) from the median of the window. The size of the sliding window was set to include the 5 nearest points on either side of the central point. Second, after removing the outliers, sigmoid curves parameterized according to the Gompertz model were fitted to the raw data of the replicates [67]. When the Gompertz model failed to provide an adequate fit, the logistic function was employed. The samples were classified as TDP-43 positive if the corresponding sigmoid curves of at least two out of three replicates reached the fluorescence threshold of 50.000 AU within 36 h. This combination of fluorescence and time thresholds was selected to achieve the greatest possible specificity while maintaining good sensitivity in the absence of a literature reference. The samples were classified as αSyn positive if the corresponding sigmoid curves of at least two out of three replicates reached the fluorescence threshold of 30.000 AU within 27 h, similar to previous studies [64, 65]. Two main parameters were extracted from the sigmoid curves of those replicates crossing the threshold (positive replicates): the last fluorescence value reached by the curve and the lag phase, which is the time required by the curve to reach the identified time-fluorescence threshold. Overall, the last fluorescence and lag phase values were computed for each TDP-43-positive subject as the median of the last fluorescence and lag phase values among the positive replicates. Likewise, overall last fluorescence and lag phase values were computed for αSyn-positive subjects.

### Evaluation of TDP-43 and NfL levels in plasma samples and eGFR calculation

Available plasma samples (45 from sporadic MND, 10 from genetic MND and 14 from CTRL subjects) were used to measure the levels of TDP-43, NfL and creatinine. Whole blood was collected in EDTA tubes after an overnight fast. The blood was centrifuged (10 min, 2500 × g at 4 °C), aliquoted and immediately stored at − 80 °C. The plasma samples were thawed for 60 min at room temperature, shaken by vortexing for 10 s and centrifuged (5 min, 10,000 × g at room temperature) before TDP-43 and creatinine level analysis. TDP-43 was measured via the Simoa TDP-43 Advantage kit and the SR-X instrument (Quanterix) according to the manufacturer’s instructions. The samples were randomized on plates and analyzed in duplicate. Serum creatinine from patients and controls was used to calculate the estimated glomerular filtration rate (eGFR) according to the modified diet in renal disease (MDRD) formula [68]. Creatinine was measured with an Abbott clinical chemistry assay and analyzed with an Architect c System 4000. To measure NfL levels, plasma samples were thawed for 30 min, vortexed for 10 s and centrifuged at 2000 × g at room temperature for 5 min. NfL levels were determined using the fully automated chemiluminescence enzyme immunoassay (CLEIA) with the Lumipulse G600II instrument and the Lumipulse G NfL Blood kit (Fujirebio).

### Statistical analysis

The lag phase and fluorescence values of TDP-43-positive subjects were compared between those with MND and those without MND via the Wilcoxon rank-sum test after checking that the normality assumption of the data did not hold with the Shapiro‒Wilk test. Univariate logistic regression analysis was performed to estimate the associations between the target variable (TDP-43_SAA positivity) and the following clinical-demographic and biological factors: sex, age at evaluation, disease duration, ongoing treatment with Riluzole, sporadic vs genetic disease, clinical phenotype, cognitive phenotype (impaired vs normal), King's staging system, rate of progression (ROP), body mass index (BMI), plasma levels of TDP-43 and NfL. Multiple logistic regression analysis was conducted if multiple factors had *p* < 0.10 in the univariate logistic regression analysis. Similarly, simple and multiple linear regression analyses were performed to evaluate the associations between the plasma levels of TDP-43 and of NfL and the aforementioned clinical-demographic factors. One-way analysis of variance (ANOVA) was used to test for differences in the means of plasma TDP-43 and NfL levels among groups according to significant categorical factors identified in the previous analysis. In addition, plasma levels of TDP-43 and of NfL were compared among sporadic MND, genetic MND and CTRLs via Wilcoxon rank-sum tests after the Shapiro‒Wilk test was used to assess normality. Statistical analyses were conducted with R (version 4.3.3); the “pracma” package was used for the Hampel filter, and the “biogrowth” package was used for fitting the Gompertz model. Statistical significance was set at p < 0.05.

## Results

### The OM of sporadic and genetic MND induces TDP-43 seeding activity

**Fig. 1:**
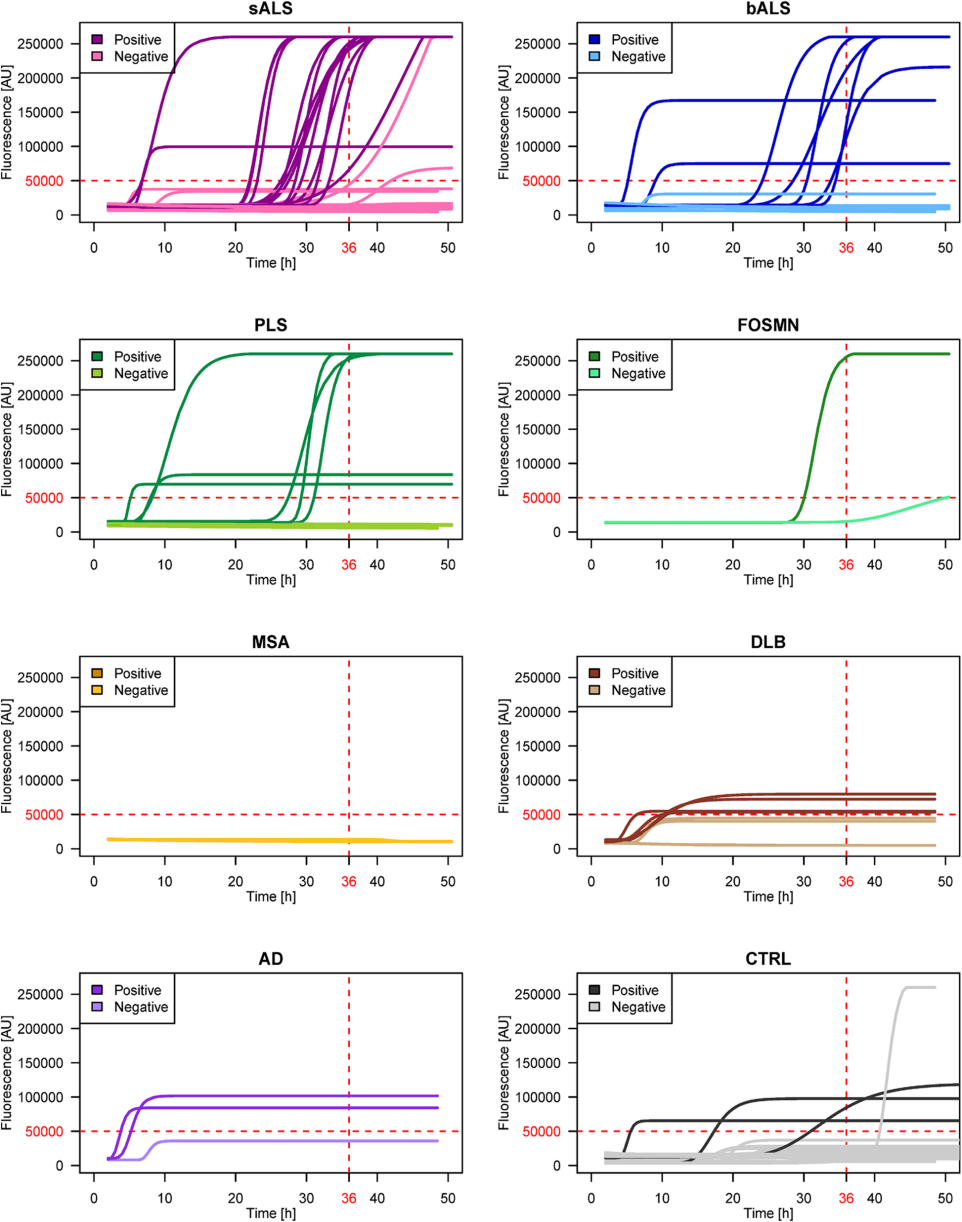
TDP-43_SAA results for OM samples from sporadic MND cases. TDP-43_SAA showed seeding activity in 43% of the sALS cases, 39% of the bALS cases, 60% of the PLS cases, 50% of the FOSMN cases, 50% of the DLB cases, and 67% of the AD cases. No seeding activity was observed in the MSA samples, whereas 14% of the CTRL samples tested positive. Abbreviations: sALS = spinal-onset amyotrophic lateral sclerosis; bALS = bulbar-onset amyotrophic lateral sclerosis; PLS = primary lateral sclerosis; FOSMN = facial onset sensory and motor neuronopathy; MSA = multiple system atrophy; DLB = dementia with Lewy body; AD = Alzheimer's disease; CTRL = controls; AU = arbitrary units; h = hours

Among sporadic MND (n = 65), TDP-43 seeding activity was observed in 15/35 sALS (43%), 7/18 bALS (39%), 6/10 PLS (60%), and 1/2 FOSMN (50%) patients. For OND cases, seeding activity was observed in 0/6 MSA (0%), 4/8 DLB (50%) and 2/3 AD (67%) samples. In the CTRL group, 3/22 (14%) samples tested positive (Fig. 1, Table 2 and Supplementary Table 1a, Additional File 1).

**Table 2:** Summary of TDP-43_SAA results

Patient group	TDP-43_SAA positive cases, % (n/N)	Lag-phase time, mean hours (SD)*	Last fluorescence value, mean AUs (SD)*
*Sporadic MND*			
sALS	43% (15/35)	23.4 (8.2)	227 933 (51 694)
bALS	39% (7/18)	23.0 (10.1)	180 887 (82 122)
PLS	60% (6/10)	18.9 (11.8)	173 091 (89 048)
FOSMN	50% (1/2)	30.5 (NA)	217 558 (NA)
*Genetic MND*			
*C9orf72*^*exp*^	50% (3/6)	27.8 (3.8)	260 000 (0)
*TARDBP*	50% (2/4)	30.2 (1.8)	234 936 (35 446)
*SQSTM1*	67% (2/3)	19.2 (18.7)	256 962 (4 295)
*C9orf72*^*exp*^ + *SQSTM1*	0% (0/1)	NA	NA
*OPTN*	100% (1/1)	20.0 (NA)	260 000 (NA)
*GLE1*	100% (1/1)	6.5 (NA)	82 886 (NA)
*FUS*	0% (0/1)	NA	NA
*SOD1*	0% (0/4)	NA	NA
*Other Neurodegenerative Diseases*			
MSA	0% (0/6)	NA	NA
AD	67% (2/3)	5.7 (1.1)	81 219 (13 158)
DLB	50% (4/8)	9.0 (1.5)	60 091 (8 448)
*CTRLs*	14% (3/22)	18.8 (12.0)	79 319 (33 479)

The average lag-phase time and fluorescence last value, stratified by clinical phenotype and genotype, are shown. *Computed over the TDP-43-positive cases only. A sample was considered positive if at least two of the three replicates reached the fluorescence threshold (50,000 AU) within 36 h. Abbreviations: MND = motor neuron disease; sALS = spinal-onset amyotrophic lateral sclerosis; bALS = bulbar-onset amyotrophic lateral sclerosis; SD = standard deviation; AU = arbitrary unit; PLS = primary lateral sclerosis; FOSMN = facial onset sensory and motor neuronopathy; *C9orf72*^*exp*^ = chromosome 9 open reading frame 72 expansion; *TARDBP* = TAR DNA-binding protein; *SQSTM1* = sequestosome 1; *OPTN *= optineurin; *GLE1* = GLE1 RNA export mediator; *FUS* = fused in sarcoma; *SOD1* = superoxide dismutase type 1; NA = not applicable; MSA = multiple system atrophy; AD = Alzheimer's disease; DLB = dementia with Lewy body; CTRLs = controls

**Fig. 2:**
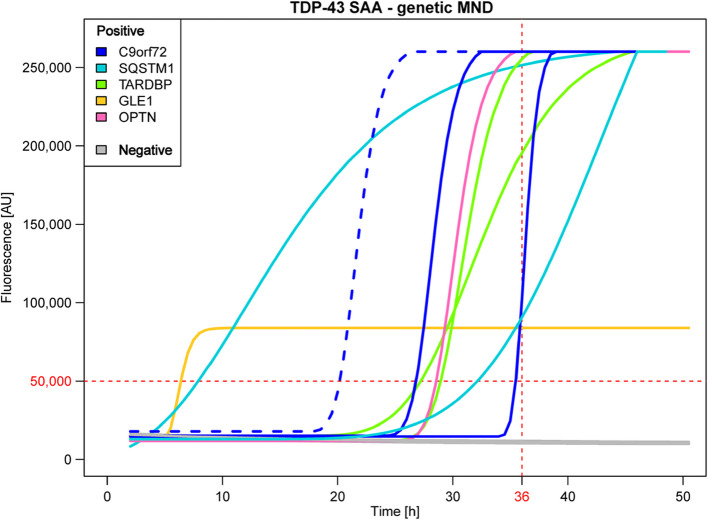
TDP-43_SAA results for OM samples from genetic MND cases. TDP-43_SAA showed seeding activity in 50% of *TARDBP* cases (green curves), 67% of *SQSTM1* cases (light blue curves), 100% of *OPTN* (pink curve) and *GLE1* cases (orange curve) and 50% of *C9orf72*^*exp*^ cases (dark blue curves) (including a presymptomatic case, dotted dark blue curve). Gray lines indicate cases in which no seeding was observed. Abbreviations: MND = motor neuron disease; *C9orf72 *= chromosome 9 open reading frame 72; *SQSTM1* = sequestosome 1; *SOD1* = superoxide dismutase type 1; *TARDBP* = TAR DNA-binding protein; *GLE1* = GLE1 RNA export mediator; *OPTN* = optineurin; AU = arbitrary units; h = hours

TDP-43 seeding was observed in 9 OM (56%) of patients with genetic MND associated with TDP-43 pathology, including 2/4 with *TARDBP* (50%), 2/3 with *SQSTM1* (67%), 1/1 with *OPTN* (100%), 1/1 with *GLE1* (100%), and 3/6 with *C9orf72*^*exp*^ (50%). Among *C9orf72*^*exp*^ carriers, one positive sample was obtained from a healthy mutation carrier who exhibited no clinical signs or symptoms of the disease at the time of sample collection and had normal cognitive efficiency but altered performance in a social cognition task (Fig. 2, Table 2 and Supplementary Table 1b, Additional File 1). Interestingly, the *GLE1* carrier tested positive for TDP-43, supporting the hypothesis that this mutation may be associated with TDP-43-related pathology. The negative results observed in the *FUS* (*n* = 1) and *SOD1* (*n* = 4) patients are in line with the evidence that TDP-43 pathology is mostly absent in these forms of familial ALS [45–47, 69].

Under our experimental conditions, the overall sensitivity of TDP-43_SAA, including sporadic and genetic MND cases, reached 46.9% (38/81), with a specificity of 88.9% (considering that 19/22 CTRLs and 5/5 genetic MND cases with *FUS* and *SOD1* mutations were negative).

**Fig. 3:**
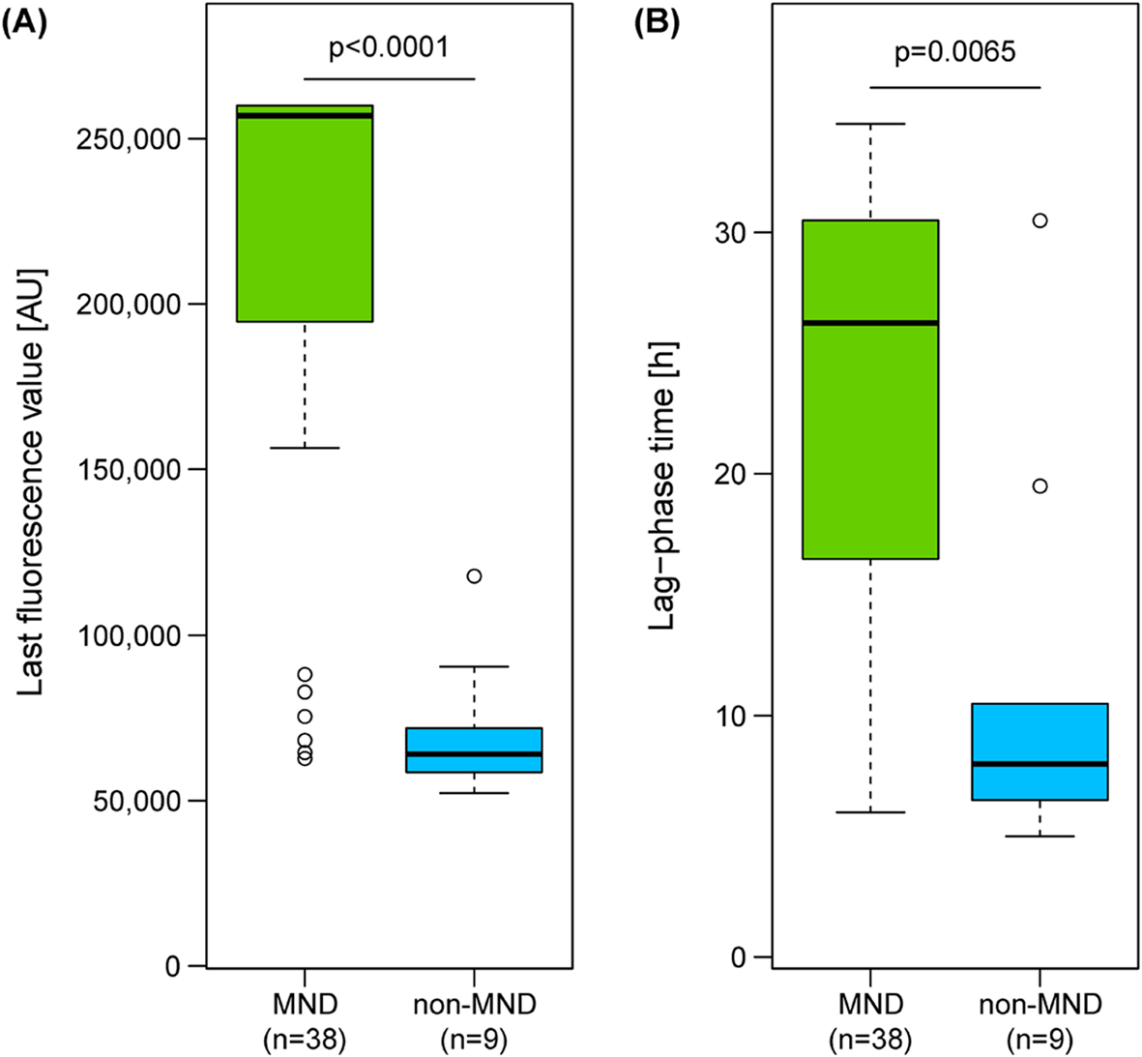
Boxplots showing the mean last fluorescence levels (A) and lag phases (B) of OM samples that tested positive for TDP-43_SAA. The average last fluorescence value observed in patients who tested positive for TDP-43_SAA (including OND patients and CTRLs, referred to as non-MND patients) was significantly greater in MND patients than in non-MND patients (**A**). Similarly, the average lag phase of MND patients was significantly longer than that of non-MND patients (**B**)

Among the samples showing TDP-43 seeding activity, the comparison of fluorescence intensity revealed a statistically significant difference (p value < 0.0001) between MND (*n* = 38) and non-MND cases (*n* = 9, including 2 AD, 4 DLB, and 3 CTRLs) (Fig. 3A and Table 2). In particular, higher mean-last fluorescence values were observed in MND patients (211,789 ± 67,679 AU) than in non-MND patients (71,195 ± 20,980 AU). In addition, lag-phase time analysis revealed a statistically significant difference (*p* = 0.0065) between MND patients and non-MND patients. As reported in the boxplot in Fig. 3B, MND patients had a longer mean lag phase than non-MND patients did (22.8 ± 9.4 h and 11.6 ± 8.3 h, respectively).

### OM of sporadic and genetic MND induces α-synuclein seeding activity in a subset of cases

**Fig. 4:**
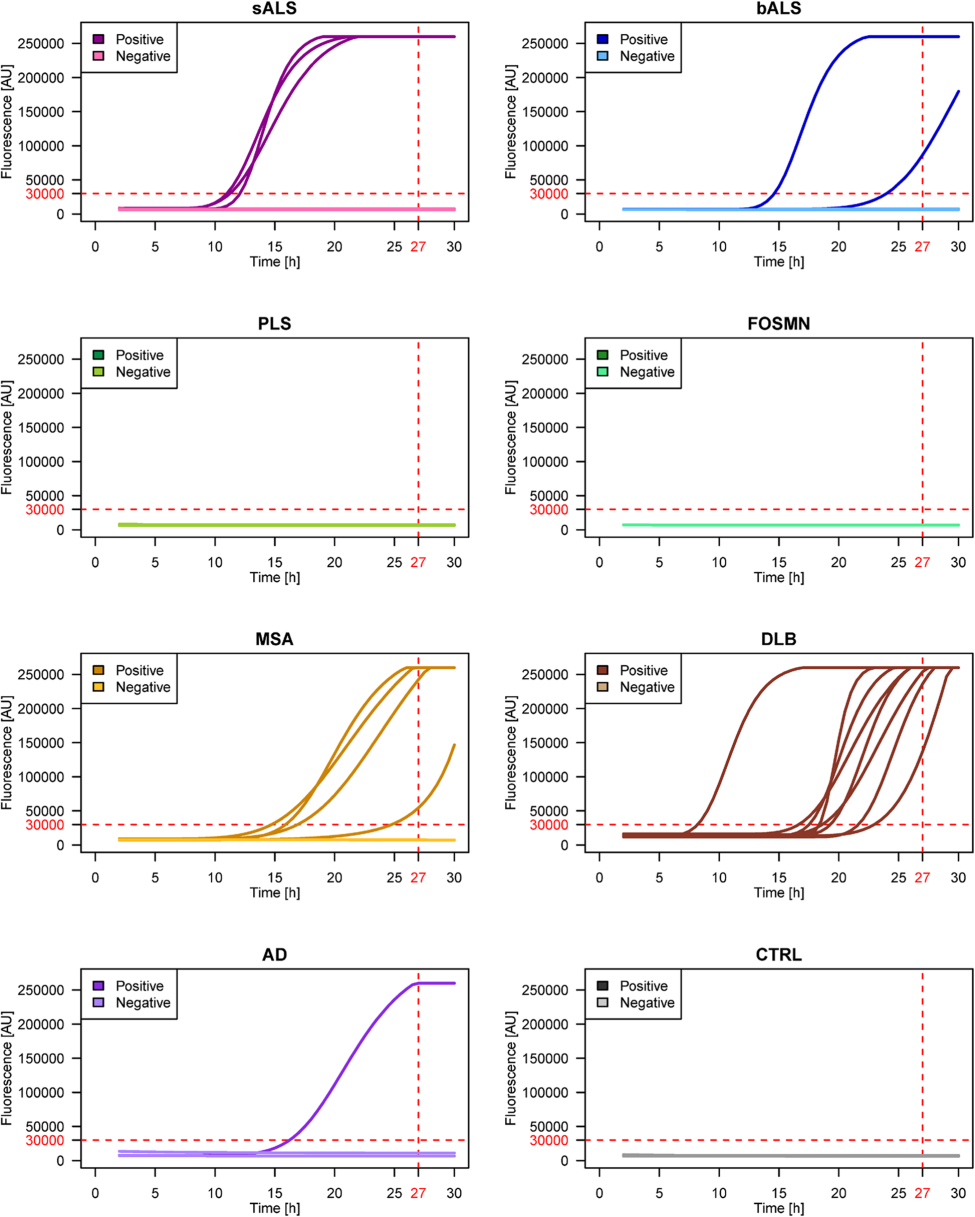
αSyn_SAA results for OM samples from sporadic MND cases. αSyn_SAA showed seeding activity in 9% of sALS cases and 11% of bALS cases, 67% of MSA cases, 100% of DLB cases and 33% of AD cases. No seeding activity was observed in the PLS, FOSMN or CTRL samples. Abbreviations: sALS = spinal-onset amyotrophic lateral sclerosis; bALS = bulbar-onset amyotrophic lateral sclerosis; PLS = primary lateral sclerosis; FOSMN = facial onset sensory and motor neuronopathy; MSA = multiple system atrophy; DLB = dementia with Lewy body; AD = Alzheimer's disease; CTRL = controls; AU = arbitrary units; h = hours

All the OM samples were subjected to αSyn_SAA analysis to test for the presence of αSyn copathology. Among sporadic MND cases, αSyn seeding activity was observed in 3 out of 35 sALS (9%) and 2 out of 18 bALS (11%) cases. No seeding activity was observed among the PLS and FOSMN cases. As expected, αSyn seeding activity was prominent in OM samples from patients with MSA (67%, 4 out of 6 cases) and DLB (100%, 8 out of 8 cases). Additionally, one out of 3 samples from an AD patient tested positive, whereas none of the CTRL samples tested positive (Fig. 4, Table 3 and Supplementary Table 1a- 1c- 1 d, Additional File 1).

**Table 3:** Summary of the αSyn_SAA results

Patient group	αSyn_SAA positive cases, % (n/N)		
	**Overall**	**Over TDP-43_SAA positive cases**	**Over TDP-43_SAA negative cases**
*Sporadic MND*			
sALS	9% (3/35)	13% (2/15)	5% (1/20)
bALS	11% (2/18)	29% (2/7)	0% (0/11)
PLS	0% (0/10)	0% (0/6)	0% (0/4)
FOSMN	0% (0/2)	0% (0/1)	0% (0/1)
*Genetic MND*			
*C9orf72*^*exp*^	17% (1/6)	0% (0/3)	33% (1/3)
*TARDBP*	25% (1/4)	0% (0/2)	50% (1/2)
*SQSTM1*	67% (2/3)	100% (2/2)	0% (0/1)
*C9orf72*^*exp*^ + *SQSTM1*	0% (0/1)	-	0% (0/1)
*OPTN*	0% (0/1)	0% (0/1)	-
*GLE1*	0% (0/1)	0% (0/1)	-
*FUS*	0% (0/1)	-	0% (0/1)
*SOD1*	0% (0/4)	-	0% (0/4)
*Other Neurodegenerative Diseases*			
MSA	67% (4/6)	0% (0/6)	67% (4/6)
AD	33% (1/3)	0% (0/2)	100% (1/1)
DLB	100% (8/8)	100% (4/4)	100% (4/4)
*CTRLs*	0% (0/22)	0% (0/3)	0% (0/19)

A sample was considered positive if at least two of the three replicates reached the fluorescence threshold (30,000 AU) within 27 h. Abbreviations: MND = motor neuron disease; sALS = spinal-onset amyotrophic lateral sclerosis; bALS = bulbar-onset amyotrophic lateral sclerosis; PLS = primary lateral sclerosis; FOSMN = facial onset sensory and motor neuronopathy; *C9orf72*^*exp*^ = chromosome 9 open reading frame 72 expansion; *TARDBP* = TAR DNA-binding protein; *SQSTM1* = sequestosome 1; *OPTN* = optineurin; *GLE1* = GLE1 RNA export mediator; *FUS* = fused in sarcoma; *SOD1* = superoxide dismutase type 1; MSA = multiple system atrophy; AD = Alzheimer's disease; DLB = dementia with Lewy body; CTRLs = controls

**Fig. 5:**
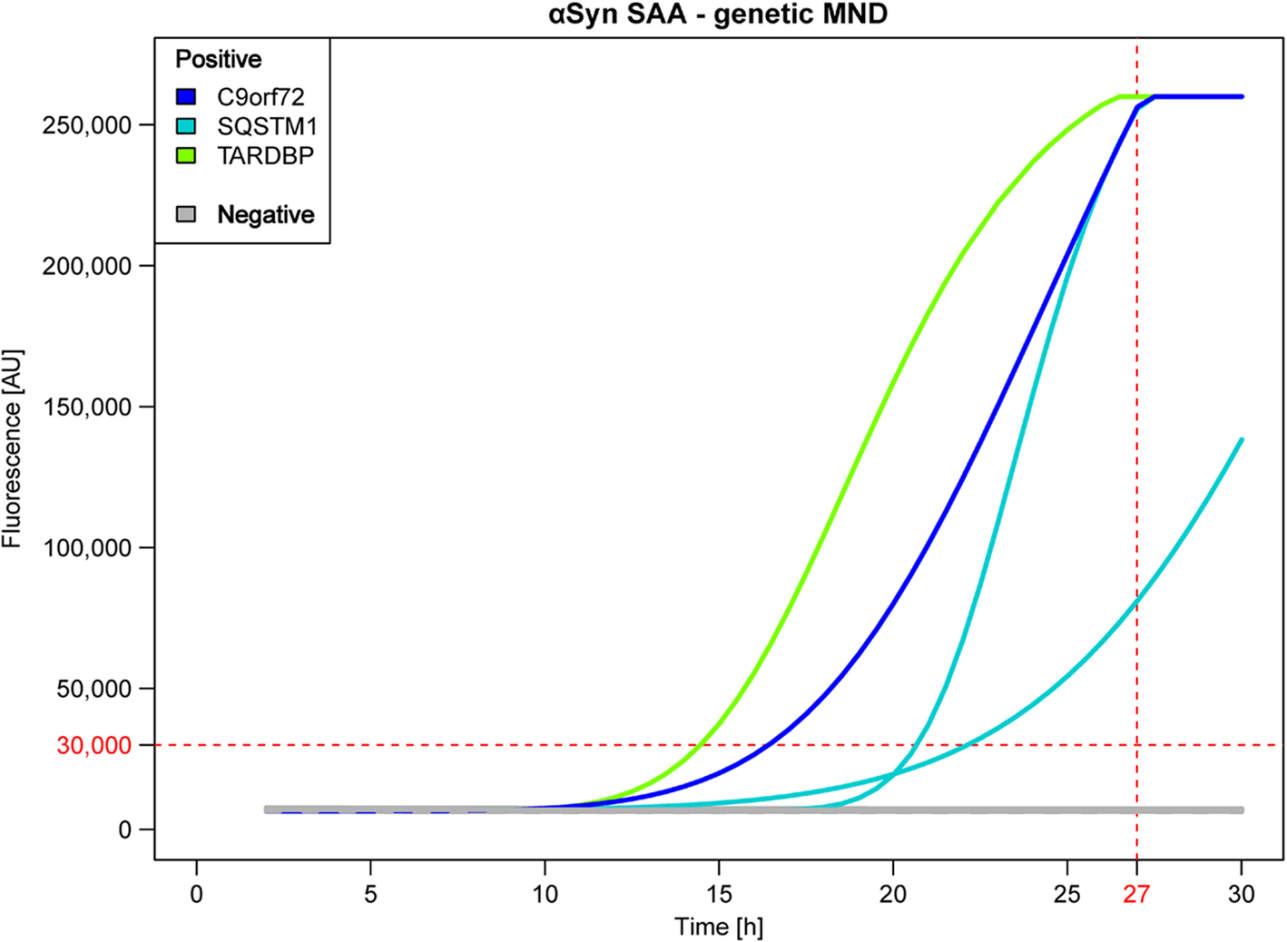
αSyn_SAA results for OM samples from genetic MND cases. αSyn_SAA showed seeding activity in 1 *C9orf72*^*exp*^ patient (dark blue curve), 2 *SQSTM1* patients (light blue curves) and 1 *TARDBP* patient (green curve). No seeding activity was detected in *C9orf72*^*exp*^ + *SQSTM1*, *OPTN*, *GLE1*, *FUS* or *SOD1* cases (gray lines). Abbreviations: MND = motor neuron disease; *C9orf72*^*exp*^ = chromosome 9 open reading frame 72 expansion; *SQSTM1* = sequestosome 1; *SOD1* = superoxide dismutase type 1; *TARDBP* = TAR DNA-binding protein; AU = arbitrary units; h = hours

Among genetic MNDs, 4 out of 21 (19%) tested positive for αSyn_SAA. Specifically, 1 out of 6 *C9orf72*^*exp*^ (17%), 2 out of 3 *SQSTM1* (67%), and 1 out of 4 *TARDBP* (25%) genes were identified. No seeding activity for αSyn was observed in patients with *C9orf72*^*exp*^ and *SQSTM1*, *OPTN*, *GLE1*, *FUS* and *SOD1* mutations (Fig. 5, Table 3 and Supplementary Table 1b, Additional File 1).

In total, we identified 9 MND samples positive for αSyn_SAA (5 sporadic and 4 genetic cases). Remarkably, 4 sporadic ALS patients (2 sALS patients and 2 bALS patients) and 2 genetic ALS patients (both *SQSTM1* carriers) were positive according to both TDP-43 and αSyn SAA analyses.

### Plasma levels of TDP-43 and NfL are increased in MND compared to controls

**Fig. 6:**
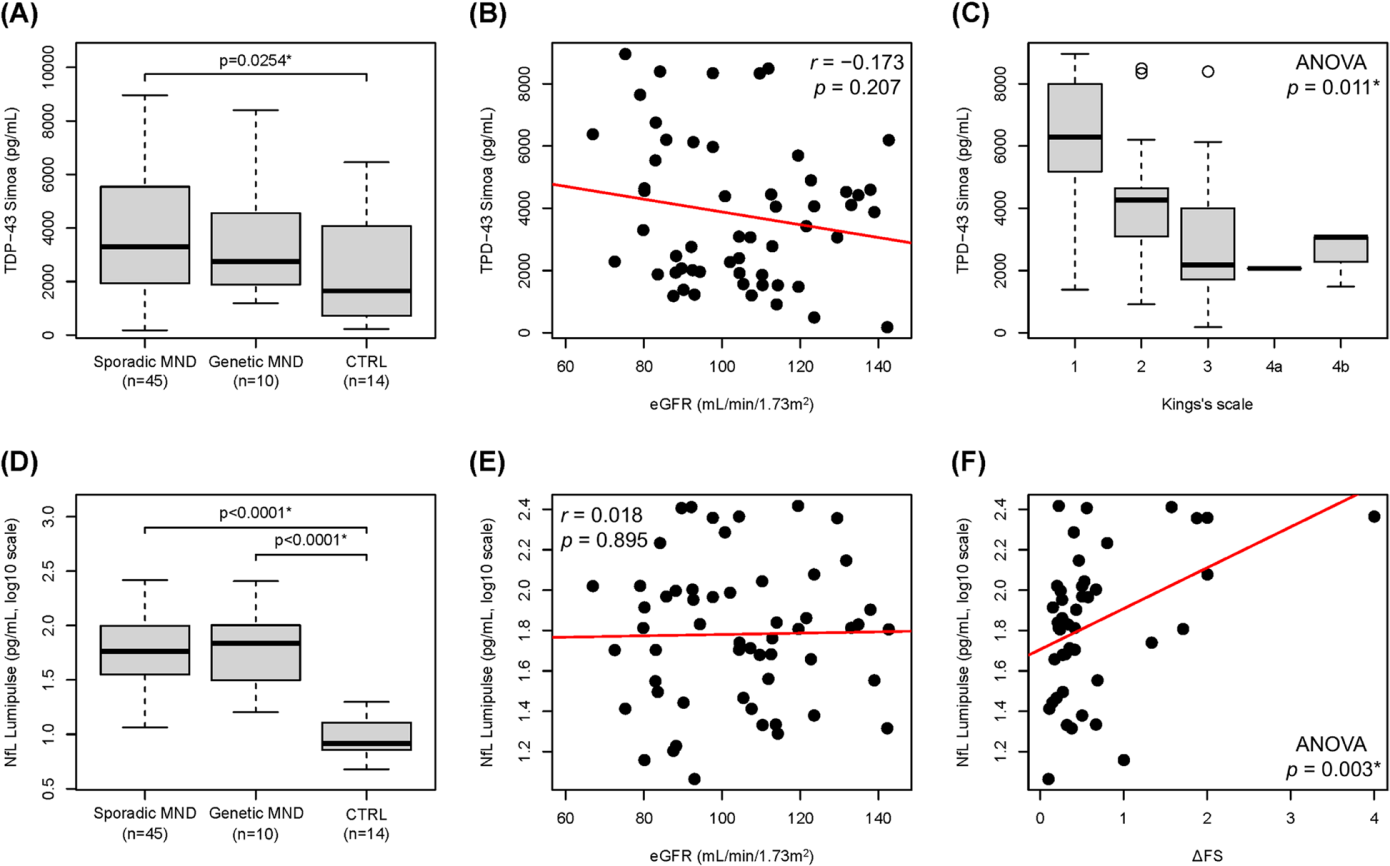
Plasma TDP-43 concentration, plasma NfL concentration, and correlation with clinical variables. Plasma TDP-43 levels in patients with sporadic MND were significantly higher compared to the CTRL group (Wilcoxon rank-sum test, *p* = 0.0254) (**A**). eGFR values were within the normal range for all patients with MND, and no statistically significant correlation was observed between eGFR values and TDP-43 levels (r = − 0.173, *p* = 0.207) (**B**). TDP-43 levels in MND patients significantly decreased with increasing disease burden, as assessed by the King's Clinical Staging System (ANOVA test, *p* = 0.011) (**C**). NfL values in patients with sporadic and genetic MND were significantly elevated compared to the CTRL group (Wilcoxon rank-sum test, both *p* < 0.0001) (**D**). No statistically significant correlation was observed between eGFR values and NfL levels (r = 0.018, *p* = 0.895) (**E**). NfL levels in MND patients significantly increased with disease progression rate (ΔFS) (ANOVA test, *p* = 0.003) (**F**)

The analysis of plasma samples revealed that patients with sporadic MND had significantly higher levels of TDP-43 compared to CTRLs (Wilcoxon test, *p* = 0.0254) (Fig. 6A). Additionally, both sporadic and genetic MND patients showed significantly higher levels of NfL than the CTRLs (Wilcoxon test, both *p* < 0.0001) (Fig. 6D). These findings agree with those of previous publications [48, 70–72]. However, in this study, we also assessed the renal function of patients by calculating the eGFR. The results confirmed that the eGFR was normal in all tested MND patients and did not significantly influence the plasma levels of TDP-43 (Pearson’s r = − 0.173, *p* = 0.207) (Fig. 6B), nor those of NfL (Pearson’s r = 0.018, *p* = 0.895) (Fig. 6E). Interestingly, we observed a statistically significant reduction in plasma TDP-43 levels in MND patients as their scores on the King’s Clinical Staging System increased (ANOVA test, *p* = 0.0112) (Fig. 6C). This suggests that as the disease progresses, there may be an increased accumulation of TDP-43 within the CNS, leading to its decreased presence in plasma. Regarding NfL, we observed a significant association with sex, with males exhibiting lower NfL levels than in females (ANOVA test, *p* = 0.0343) (Supplementary Table 3, Additional File 3). Interestingly, plasma NfL levels showed a positive correlation with disease progression rate (ΔFS) (ANOVA test, *p* = 0.0032) (Fig. 6F) and a negative correlation with the ALS-specific ECAS score (ANOVA test, *p* = 0.0090) (Supplementary Table 3, Additional File 3).

Given the normal renal function of these patients, we can hypothesize that these alterations may be directly associated with the disease itself. Statistical analysis confirmed the lack of significant correlations between TDP-43 and NfL levels with other demographic and clinical parameters (Supplementary Table 2, Additional File 2; Supplementary Table 3, Additional File 3).

### Lack of significant correlations between TDP-43_SAA and clinical, neuropsychological and biological parameters in MND patients

Logistic regression analysis did not reveal any statistically significant correlation between the SAA results for TDP-43 and other clinical parameters investigated, including sex, patient age, BMI, clinical genotype/phenotype, cognitive phenotype, ECAS scores, disease duration, TDP-43 plasma levels, NfL plasma levels, progression rate and treatment with Riluzole (Table 4).

**Table 4:** Logistic regression results for the associations of TDP-43 positivity with the clinical, demographic and biological characteristics of patients with MND

Clinical parameter	Group	TDP-43_SAA result		Univariate OR (95% CI, *p* value)
		*Negative*	*Positive*	
Sex	Female	18 (64.3%)	10 (35.7%)	ref
	Male	30 (51.7%)	28 (48.3%)	1.680 (0.672–4.367, *p* = 0.2737)
Age, years	*Mean (SD)*	61.0 (10.3)	63.1 (9.8)	1.021 (0.979–1.068, *p* = 0.3357)
BMI, kg/m^2^	*Mean (SD)*	23.9 (3.4)	24.8 (3.9)	1.077 (0.952–1.228, *p* = 0.2470)
Genotype	Genetic	12 (57.1%)	9 (42.9%)	ref
	Sporadic	36 (55.4%)	29 (44.6%)	1.074 (0.399–2.965, *p* = 0.8878)
Clinical phenotype*	sALS	29 (60.4%)	19 (39.6%)	ref
	bALS	14 (58.3%)	10 (41.7%)	1.090 (0.396–2.950, *p* = 0.8651)
	PLS	4 (36.4%)	7 (63.6%)	2.671 (0.708–11.391, *p* = 0.1562)
	FOSMN	1 (50.0%)	1 (50.0%)	1.526 (0.058–40.182, *p* = 0.7698)
Cognitive phenotype	Normal	25 (55.6%)	20 (44.4%)	ref
	Impaired	17 (56.7%)	13 (43.3%)	0.956 (0.373–2.427, *p* = 0.9243)
ECAS, ALS-specific	*Mean (SD)*	68.8 (11.8)	69.3 (13.6)	1.003 (0.965–1.044, *p* = 0.8703)
ECAS, non-ALS specific	*Mean (SD)*	26.0 (4.5)	25.4 (4.3)	0.970 (0.867–1.082, *p* = 0.5826)
ECAS, total	*Mean (SD)*	94.7 (14.5)	94.6 (16.1)	1.000 (0.968–1.033, *p* = 0.9822)
Disease duration, months	*Mean (SD)*	29.9 (29.1)	35.2 (32.5)	1.006 (0.991–1.021, *p* = 0.4326)
King’s clinical staging	1	7 (53.8%)	6 (46.2%)	ref
	2	20 (69.0%)	9 (31.0%)	0.525 (0.134–2.046, *p* = 0.3476)
	3	12 (60.0%)	8 (40.0%)	0.778 (0.186–3.239, *p* = 0.7269)
	4a	2 (66.7%)	1 (33.3%)	0.583 (0.024–7.681, *p* = 0.6887)
	4b	2 (33.3%)	4 (66.7%)	2.333 (0.328–21.558, *p* = 0.4104)
ΔFS	*Mean (SD)*	0.6 (0.7)	0.6 (0.6)	1.031 (0.458–2.215, *p* = 0.9358)
Rate of progression	Slow	26 (61.9%)	16 (38.1%)	ref
	Intermediate	9 (60.0%)	6 (40.0%)	1.083 (0.312–3.595, *p* = 0.8965)
	Fast	7 (53.8%)	6 (46.2%)	1.393 (0.386–4.941, *p* = 0.6050)
Riluzole treatment	Absent	32 (54.2%)	27 (45.8%)	ref
	Present	16 (59.3%)	11 (40.7%)	0.815 (0.318–2.039, *p* = 0.6636)
TDP-43 Simoa, pg/ml	*Mean (SD)*	3 958.2 (2 472.2)	3 565.8 (2 098.5)	1.000 (1.000–1.000, *p* = 0.5360)
NfL Lumipulse	*Mean (SD)*	79.9 (66.8)	87.0 (72.8)	1.002 (0.993–1.010, *p* = 0.7067)

^*^Regardless of the genotype. The data reported in the “TDP-43 result” columns are the number of patients and, in parenthesis, percentages, unless otherwise specified. Abbreviations: ref = reference level; SD = standard deviation; OR = odds ratio; CI = confidence interval; BMI = body mass index; sALS = spinal-onset amyotrophic lateral sclerosis; bALS = bulbar-onset amyotrophic lateral sclerosis; PLS = primary lateral sclerosis; FOSMN = facial onset sensory and motor neuronopathy; ECAS = Edinburgh Cognitive and Behavioral ALS Screen; ΔFS = disease progression rate

## Discussion

Since the first reported evidence in 2006, TDP-43 aggregates have been described as a key pathological feature of various neurodegenerative disorders, collectively known as TDP-43 proteinopathies [73]. Although the exact role of these aggregates in pathology has not yet been fully elucidated, the prion-like behavior of TDP-43 has generated increasing interest. This characteristic has been exploited to develop SAA, enabling the detection of pathological TDP-43 in CSF and OM samples [54, 55]. Hence, TDP- 43_SAA is emerging as a promising tool for diagnosing TDP-43 proteinopathies using easily accessible tissues. Here, we provide proof-of-concept that TDP-43 seeding can be efficiently detected in the OM of patients with different forms of genetic and sporadic MND, regardless of their clinical phenotype and genetic profile. The current ALS pathological staging system places the olfactory region involvement as a late event (Braak stage 4), yet previous studies have found TDP-43-positive inclusions in olfactory centers of 30–50% of postmortem ALS brains [51, 74]. Specifically, TDP-43 pathology was more abundant in secondary olfactory regions and least severe in peripheral areas such as the olfactory bulb, suggesting a centrifugal progression of TDP-43 pathology, unlike the centripetal spread of αSyn proposed for PD [75]. These findings may help to explain the limited sensitivity of our TDP-43_SAA, considering the peripheral location of the OM. The lack of correlation between TDP-43_SAA positivity and disease duration also suggests that TDP-43 seeding can be detected in early, pre-clinical stages, as observed in the only pre-symptomatic carrier enrolled in our cohort. Although the assessment of further presymptomatic subjects is needed to confirm this assumption, similar results have already been reported for CSF TDP-43_SAA in the granulin (*GRN)* healthy carrier included in the study by Scialò and collaborators [55]. The most compelling finding of our study is that OM samples from patients with sporadic MND (sALS, bALS, PLS, and FOSMN) tested positive for TDP-43_SAA, as did some genetic ALS cases, all of which are associated with TDP-43 pathology. Interestingly, genetic forms of ALS not associated with TDP-43 pathology (*FUS* and *SOD1*) did not induce TDP-43 seeding activity. However, these observations are based on a limited number of genetic samples, and further studies with a larger cohort could provide additional insights. Notably, we unexpectedly observed TDP-43 seeding activity in the OM of patients with DLB, AD and even in healthy individuals. This likely points to the presence of coexisting pathologies or early, subclinical stages of the disease. TDP-43 aggregates have been found in the brains of patients with MSA (7%), DLB (56%), AD (57%), and even elderly healthy individuals (up to 23%, with increasing incidence with age) [24]. These observations align with our TDP-43_SAA results in OM samples from OND patients and CTRLs, and raise the possibility that misfolded TDP-43 could potentially serve as a biomarker for neurodegenerative processes linked to dementia. Interestingly, the TDP-43 aggregation curves derived from OM analyses of the OND and CTRL samples exhibit distinctive features. Specifically, the fluorescence values for these curves remain notably low compared with those observed in OM analyses of patients with sporadic or genetic MND. This unique pattern highlights a significant difference in TDP-43 aggregation behavior across these groups. However, further prospective studies specifically designed to evaluate the clinical utility of this parameter as a diagnostic biomarker should be performed. On the contrary, explaining why the OM of a subset of sporadic and genetic MND patients tested negative for TDP-43_SAA remains challenging. It is possible that these specific cases are associated with different TDP-43 strains. Alternatively, pathological TDP-43 may not have reached the OM, or the TDP-43 strains present may lack the ability to promote aggregation in vitro, leading to a negative TDP-43_SAA result. Furthermore, we cannot rule out the possibility that preanalytical treatment of OM altered the seeding properties of these specific TDP-43 strains, contributing to the negative TDP-43_SAA results. Interestingly, we did not observe any significant correlation between the TDP-43_SAA findings and the other biochemical, clinical or neuropsychological parameters considered. In a previous publication, TDP-43 seeding activity in OM samples from FTD patients appeared to be correlated with the presence of dementia [54]. In this study, we did not observe a statistically significant correlation between the presence of dementia and the ALS samples tested. However, the high TDP-43 seeding activity observed in the AD and DLB samples included in our study supports the plausibility of this association. αSyn is also emerging as a potential pathogenic contributor to ALS [76]. Studies have shown that αSyn copathology occurs in 11% of ALS cases and αSyn oligomers promote TDP-43 seeding in vitro (cross-seeding effect), suggesting that these proteins may synergistically interact [77, 78]. Recently, αSyn_SAA analyses detected pathological αSyn in the CSF of patients with sporadic or familial ALS (18/127 tested samples), and notably, αSyn_SAA analyses of our OM samples is consistent with these CSF findings [77]. However, interpreting these results remains complex, as certain TDP-43 strains that fail to promote TDP-43 aggregation, may induce αSyn aggregation, adding complexity to the findings. Neither the αSyn_SAA findings nor the combination of TDP-43_SAA and αSyn_SAA results showed any correlation with the other biochemical, clinical or neuropsychological parameters analyzed. This lack of association underscores the current complexity of linking these biomarkers to clinical manifestations. Finally, our data must consider that olfactory neurons renew every 2–3 months, which could influence the results of SAA analyses for TDP-43 and αSyn [79]. Simoa analysis confirmed significantly higher plasma levels of TDP-43 in sporadic MND patients than in controls, independent of eGFR values. Interestingly, as the disease progresses, a reduction in plasma TDP-43 levels is observed, suggesting that intracerebral accumulation of the protein may ultimately influence its plasma TDP-43 concentration. Similarly, Lumipulse analysis confirmed significantly higher plasma levels of NfL in both sporadic and genetic MND, confirming once more the good accuracy of this biomarker in discriminating MND patients from healthy controls [80]. Also, we observed significantly higher plasma NfL levels in female, fast progressing disease, and MND with more severe cognitive impairment considering both total ECAS score and ALS-specific domains. Previous studies have already reported similar findings regarding sex difference and the positive correlation between NfL levels and disease progression rate [81–85], which is not surprising considering that NfL is largely released when neuroaxonal damage and degeneration occur. To our knowledge, the positive correlation between plasma NfL levels and the severity of cognitive impairment has not been previously reported, with some evidences finding no significant association between these two variables [80]; however, this field certainly requires further investigation on larger patient cohorts and it is beyond the scope of the present study. Our data provide new perspectives on ALS dynamics and pathophysiology. They also suggest that SAA for both TDP-43 and αSyn, along with plasma TDP-43 and plasma NfL could serve as promising biomarkers for monitoring disease progression and as valuable tools for patient stratification, challenging previous assumptions in the field.

## Conclusions

Our work demonstrates for the first time that TDP-43 seeding activity is detectable in the OM of patients with sporadic and some genetic forms of ALS. In addition, plasma TDP-43 and NfL levels can be exploited for future diagnostic and intervention studies. Beyond the biological significance of these innovative findings, which could help us to better understand the pathophysiological processes of ALS and eventually recognize disease phenotypes, the clinical utility of OM TDP-43_SAA analysis in a diagnostic setting needs to be further explored. By combining SAA analyses for both TDP-43 and αSyn, we identified αSyn copathology in a small subset of ALS patients. Assessing whether these findings truly reflect CNS pathology is crucial. Although we cannot confirm copathologies postmortem, as all patients included in this study are alive, this remains an important area of investigation to better understand the link between peripheral pathology and CNS involvement in ALS. In addition to the inability to directly study the neuropathology of ALS patients, we recognize that our study is based on a small sample size, underscoring the need for further analysis with a larger cohort. Ultimately, our primary aim was to explore whether integrating these tests with traditional clinical, instrumental, and laboratory analyses could create a biological fingerprint capable of distinguishing between ALS phenotypes, especially in the early stages of the disease. While our results have not yet fully achieved this goal, we are confident that further improvements in experimental SAA protocols for analyzing OM or other tissues (e.g., CSF, skin, saliva, and urine/blood-derived EVs) will enhance the sensitivity of TDP-43_SAA, eventually leading to better disease characterization. By integrating these assays (SAA, NfL and TDP-43 assessment) with conventional diagnostic tests, we can hopefully move closer to developing precise biological fingerprints of ALS to overcome the current limitations of clinically driven diagnosis [50]. With continuous refinement, these innovative tools promise to increase diagnostic accuracy, patient stratification, and efficacy in clinical trials, ultimately improving outcomes for ALS patients.

## Supplementary Information

Supplementary Material 1. Table S1. Comparison of SAA analyses and characteristics of the study population. The results of SAA analysis for TDP-43 and αSyn are listed for each subject: Sporadic MND, Genetic MND, Other neurodegenerative disorders, Controls. Abbreviations: POS = positive by SAA; NEG = negative by SAA; ID = identification; NA = not applicable; M = male; F = female; FOSMN = facial onset sensory and motor neuronopathy; bALS = bulbar-onset amyotrophic lateral sclerosis; sALS = spinal-onset amyotrophic lateral sclerosis; PLS = primary lateral sclerosis; MSA = multiple system atrophy; DLB = dementia with Lewy body; AD = Alzheimer's disease; CTRL = controls; ALS-FTD = ALS with frontotemporal dementia; ALScn = cognitively normal ALS; ALSci = ALS with cognitive impairment; ALSbi = ALS with behavioral impairment; ALScbi = ALS with combined cognitive and behavioral impairment; ALSnex = ALS with nonexecutive dysfunction; y = years; m = months; ALSFRSr_tot = ALS Functional Rating Scale revised total score; ΔFS = disease progression rate, calculated according to the following formula:/[disease duration at evaluation], as reported in Methods; BMI = body mass index; *TARDBP* = TAR DNA binding protein; *C9orf72*^*exp*^ = Chromosome 9 open reading frame 72 expansion; *SQSTM1*= sequestosome 1; *OPTN* = optineurin; *GLE1* = GLE1 RNA export mediator; het = heterozygous; hom = homozygous

Supplementary Material 2. Table S2. Correlations between plasma TDP-43 levels and other clinical parameters. Except for King’s clinical staging, no significant correlations were observed between plasma TDP-43 levels and the parameters listed in the table below. Abbreviations: ref = reference level; CI = confidence interval; BMI = body mass index; sALS = spinal-onset amyotrophic lateral sclerosis; bALS = bulbar-onset amyotrophic lateral sclerosis; PLS = primary lateral sclerosis; ECAS = Edinburgh Cognitive and Behavioral ALS Screen; ΔFS = disease progression rate; eGFR = estimated glomerular filtration rate

Supplementary Material 3. Table S3. Correlations between plasma NfL levels and other clinical parameters. Sex, age, and those variables with *p*< 0.10 at univariable analysis were included in the multivariable linear regression model, except for “Rate of progression” that was significantly correlated with ΔFSand therefore redundant. Abbreviations: NfL = neurofilament-light chain; ref = reference level; CI = confidence interval; BMI = body mass index; sALS = spinal-onset amyotrophic lateral sclerosis; bALS = bulbar-onset amyotrophic lateral sclerosis; PLS = primary lateral sclerosis; ECAS = Edinburgh Cognitive and Behavioral ALS Screen; ΔFS = disease progression rate; eGFR = estimated glomerular filtration rate.

## Data Availability

The datasets used and analysed during the current study are available from the corresponding author on reasonable request.

## Abbreviations

AD: Alzheimer’s disease
ALS: Amyotrophic lateral sclerosis
ALS-FTD: ALS with frontotemporal dementia
ALSnex: ALS with nonexecutive dysfunction
ALSbi: ALS with behavioral impairment
ALScbi: ALS with combined cognitive and behavioral impairment
ALSci: ALS with cognitive impairment
ALScn: Cognitively normal ALS
ALSFRS-R: ALS Functional Rating Scale Revised
AU: Arbitrary units
bALS: Bulbar-onset ALS
*C9orf72*^*exp*^: Chromosome 9 open reading frame 72 expansion
CBD: Corticobasal degeneration
CJD: Creutzfeldt–Jakob disease
CNS: Central nervous system
CSF: Cerebrospinal fluid
CTFs: C-terminal fragments
ddH2O: Bidistilled water
DLB: Dementia with Lewy bodies
ECAS: Edinburgh Cognitive and Behavioral ALS Screen
EDTA: Ethylenediaminetetraacetic acid
eGFR: Estimated glomerular filtration rate
EVs: Extracellular vesicles
FOSMN: Facial onset sensory and motor neuronopathy
FTD: Frontotemporal dementia
FTLD-TDP: Frontotemporal lobar degeneration with TPD-43-immunoreactive pathology
*FUS*: Fused in sarcoma
GdnHCl: Guanidine hydrochloride
*GLE1*: GLE1 RNA export mediator
*GRN*: Granulin
HD: Huntington’s disease
HuTDP-43(263–414): Recombinant human truncated TDP-43
LATE: Limbic-predominant age-related TDP- 43 encephalopathy
LMNs: Lower motor neurons
MDRD: Modification of diet in renal disease
MND: Motor neuron disease
MSA: Multiple system atrophy
NCIs: Neuronal cytoplasmic inclusions
NGS: Next-generation sequencing
NfL: Neurofilament light chain
OM: Olfactory mucosa
OND: Other neurodegenerative disorders
*OPTN*: Optineurin
OR: Odds ratio
PD: Parkinson’s disease
PLS: Primary lateral sclerosis
PMA: Progressive muscular atrophy
PSP: Progressive supranuclear palsy
rALS: Respiratory-onset ALS
rec-αSyn: Recombinant human α-Syn
ROP: Rate of progression
SAA(s): Seed amplification assay(s)
sALS: Spinal-onset ALS
SD: Standard deviation
SDS: Sodium dodecyl sulfate
sIBM: Sporadic inclusion body myositis
*SOD1*: Superoxide dismutase type 1
*SQSTM1*: Sequestosome 1
*TARDBP*: TAR DNA binding protein
*TBK1*: TANK binding kinase 1
TDP-43: Transactive response DNA-binding protein 43 kDa
TDP-43_SAA: TDP-43 seed amplification assay
ThT: Thioflavin-T
*UBQLN2*: Ubiquilin- 2
UMNs: Upper motor neurons
αSyn: α-Synuclein
αSyn_SAA: α-Synuclein seed amplification assay
ΔFS: Disease progression rate
