# Supplementary material for: TDP-43 seeding activity in the olfactory mucosa of patients with amyotrophic lateral sclerosis
Source: Mol Neurodegener. 2025 Apr 26;20:49. doi: 10.1186/s13024-025-00833-0 (PMC12034174; doi:10.1186/s13024-025-00833-0)
Supplement: Supplementary file 2 — Supplementary Material 2. Table S2. Correlations between plasma TDP-43 levels and other clinical parameters. Except for King’s clinical staging, no significant correlations were observed between plasma TDP-43 levels and the parameters listed in the table below. Abbreviations: ref = reference level; CI = confidence interval; BMI = body mass index; sALS = spinal-onset amyotrophic lateral sclerosis; bALS = bulbar-onset amyotrophic lateral sclerosis; PLS = primary lateral sclerosis; ECAS = Edinburgh Cognitive and Behavioral ALS Screen; ΔFS = disease progression rate; eGFR = estimated glomerular filtration rate [file 13024_2025_833_MOESM2_ESM.pdf]

Supplementary Table 2.

Correlations between plasma TDP-43 levels and other clinical parameters. Except for King's clinical staging, no significant correlations were observed between plasma TDP-43 levels and the parameters listed in the table below.

Factor	Group	Beta coefficient (95% CI, p value)
Sex	Female	ref
	Male	-135.022 (-1555.260 to 1285.217, p=0.8495)
Age, years		43.965 (-22.997 to 110.927, p=0.1935)
BMI, kg/m^2		104.025 (-65.259 to 273.308, p=0.2230)
Genotype	Genetic	ref
	Sporadic	320.914 (-1317.214 to 1959.042, p=0.6959)
Clinical phenotype†	sALS	ref
	bALS	353.818 (-1037.698 to 1745.335, p=0.6119)
	PLS	-1019.759 (-2998.100 to 958.582, p=0.3056)
Cognitive phenotype	Normal	ref
	Impaired	-248.868 (-1610.784 to 1113.047, p=0.7149)
ECAS, ALS-specific		16.995 (-48.072 to 82.061, p=0.6011)
ECAS, non-ALS specific		87.767 (-87.292 to 262.827, p=0.3178)
ECAS, total		19.547 (-34.527 to 73.621, p=0.4700)
Disease duration, months		-10.318 (-32.340 to 11.703, p=0.3514)
King's clinical staging	1	ref
	2	-1881.809 (-3699.182 to -64.435, p=0.0428)*
	3	-3222.001 (-5073.994 to -1370.007, p=0.0011)*
	4b	-3617.686 (-6513.225 to -722.146, p=0.0156)*
ΔFS		-410.478 (-1369.947 to 548.992, p=0.3930)
Rate of progression	Slow	ref
	Intermediate	90.393 (-1587.790 to 1768.576, p=0.9140)

	Fast	-869.928 (-2756.308 to 1016.453, p=0.3573)
Riluzole treatment	Absent	ref
	Present	-1265.473 (-2757.281 to 226.335, p=0.0947)
eGFR		-20.529 (-52.781 to 11.723, p=0.2073)

†Regardless of the genotype. *Statistically significant ($p < 0.05$).

Abbreviations: ref = reference level; CI = confidence interval; BMI = body mass index; sALS = spinal-onset amyotrophic lateral sclerosis; bALS = bulbar-onset amyotrophic lateral sclerosis; PLS = primary lateral sclerosis; ECAS = Edinburgh Cognitive and Behavioral ALS Screen; ΔFS = disease progression rate; eGFR = estimated glomerular filtration rate.
