# Supplementary material for: TDP-43 seeding activity in the olfactory mucosa of patients with amyotrophic lateral sclerosis
Source: Mol Neurodegener. 2025 Apr 26;20:49. doi: 10.1186/s13024-025-00833-0 (PMC12034174; doi:10.1186/s13024-025-00833-0)
Supplement: Supplementary file 3 — Supplementary Material 3. Table S3. Correlations between plasma NfL levels and other clinical parameters. Sex, age, and those variables with p< 0.10 at univariable analysis were included in the multivariable linear regression model, except for “Rate of progression” that was significantly correlated with ΔFSand therefore redundant. Abbreviations: NfL = neurofilament-light chain; ref = reference level; CI = confidence interval; BMI = body mass index; sALS = spinal-onset amyotrophic lateral sclerosis; bALS = bulbar-onset amyotrophic lateral sclerosis; PLS = primary lateral sclerosis; ECAS = Edinburgh Cognitive and Behavioral ALS Screen; ΔFS = disease progression rate; eGFR = estimated glomerular filtration rate. [file 13024_2025_833_MOESM3_ESM.pdf]

Supplementary Table 3.

Correlations between plasma NfL levels and other clinical parameters. Sex, age, and those variables with p<0.10 at univariable analysis were included in the multivariable linear regression model, except for "Rate of progression" that was significantly correlated with ΔFS (ANOVA p<0.0001) and therefore redundant.

**Factor**	**Group**	**Beta coefficient (95% CI, p-value)**	
		**Univariable model**	**Multivariable model**
Sex	Female	ref	ref
	Male	-39.054 (-79.757 to 1.649, p=0.0597)	-47.107 (-90.497 to -3.717, p=0.0343)*
Age, years		0.987 (-1.011 to 2.985, p=0.3263)	0.497 (-2.100 to 3.094, p=0.6993)
BMI, kg/m^2		-1.668 (-6.835 to 3.499, p=0.5198)	-
Genotype	Genetic	ref	-
	Sporadic	-6.459 (-55.041 to 42.122, p=0.7908)	-
Clinical phenotype†	sALS	ref	-
	bALS	-3.666 (-44.578 to 37.246, p=0.8579)	-
	PLS	-46.911 (-105.076 to 11.253, p=0.1116)	-
Cognitive phenotype	Normal	ref	-
	Impaired	1.747 (-39.801 to 43.296, p=0.9330)	-
ECAS, ALS-specific		-1.715 (-3.666 to 0.236, p=0.0834)	-2.762 (-4.785 to -0.739, p=0.0090)*
ECAS, non-ALS specific		-1.120 (-6.602 to 4.361, p=0.6825)	-
ECAS, total		-1.282 (-2.920 to 0.357, p=0.1220)	-
Disease duration, months		-0.895 (-1.530 to -0.260, p=0.0066)	-0.732 (-1.632 to 0.169, p=0.1076)
King's clinical staging	1	ref	-
	2	-4.125 (-63.819 to 55.569, p=0.8897)	-
	3	11.511 (-49.320 to 72.342, p=0.7043)	-
	4b	31.808 (-63.299 to 126.916, p=0.5032)	-
ΔFS		50.277 (24.051 to 76.503, p=0.0004)	45.706 (16.516 to 74.895, p=0.0032)*
Rate of progression	Slow	ref	-

	Intermediate	20.043 (-29.017 to 69.104, p=0.4143)	-
	Fast	76.265 (21.117 to 131.412, p=0.0079)	-
Riluzole treatment	Absent	ref	-
	Present	-28.445 (-73.162 to 16.272, p=0.2076)	-
eGFR		-0.044 (-1.015 to 0.926, p=0.9273)	-
Plasma TDP-43 levels		0.005 (-0.003 to 0.013, p=0.2548)	-

†Regardless of the genotype. *Statistically significant (p < 0.05).

Abbreviations: NfL = neurofilament-light chain; ref = reference level; CI = confidence interval; BMI = body mass index; sALS = spinal-onset amyotrophic lateral sclerosis; bALS = bulbar-onset amyotrophic lateral sclerosis; PLS = primary lateral sclerosis; ECAS = Edinburgh Cognitive and Behavioral ALS Screen; ΔFS = disease progression rate; eGFR = estimated glomerular filtration rate.
